# The use of pre-operative imaging and intraoperative parathyroid hormone level to guide surgical management of tertiary hyperparathyroidism from X-linked hypophosphatemic rickets: a case report

**DOI:** 10.4076/1757-1626-2-7572

**Published:** 2009-09-10

**Authors:** Matthew D Neal, Berthony Deslouches, Jennifer Ogilvie

**Affiliations:** Division of Endocrine Surgery, Department of Surgery, University of Pittsburgh Medical Center200 Lothrop Street, Pittsburgh, PA 15213USA

## Abstract

**Introduction:**

To describe the use of combined preoperative imaging and intraoperative parathyroid hormone as a novel approach in the surgical management of a patient with tertiary hyperparathyroidism associated with X-linked hypophosphatemic rickets.

**Case presentation:**

We present the first documented description of combined preoperative imaging and intraoperative parathyroid hormone as well as a review of the literature surrounding the surgical management of tertiary hyperparathyroidism in the setting of X-linked hypophosphatemic rickets.

A 23 year-old female with X-linked hypophosphatemic rickets and renal impairment presented with symptomatic hypercalcemia and tertiary hyperparathyroidism. She had failed medical management and presented for surgical evaluation. Technitium-99 m Sestamibi SPECT imaging and parathyroid ultrasound were used to localize the enlarged parathyroid glands preoperatively. Intraoperative findings correlated well with pre-operative imaging. She underwent successful subtotal parathyroidectomy for four-gland hyperplasia, using intraoperative parathyroid hormone guidance. Despite severe post-operative bone hunger, her serum calcium normalized and she experienced resolution of her preoperative symptoms.

**Conclusion:**

X-linked hypophosphatemic rickets is an uncommon disorder of phosphate metabolism resulting in bone deformity. Patients are predisposed to the development of secondary hyperparathyroidism due to chronic vitamin D supplementation which may progress to tertiary hyperparathyroidism with autonomous parathyroid function. Preoperative evaluation with Technitium-99 m Sestamibi SPECT and ultrasound imaging, as well as the use of intraoperative parathyroid hormone are effective in guiding surgical resection. Subtotal parathyroidectomy with cryopreservation is indicated to produce operative cure and limit the risk of recurrence. Although these patients are susceptible to severe postoperative bone hunger, appropriate supplementation with intravenous and oral calcium can minimize hypocalcemic symptoms.

## Introduction

X-linked hypophosphatemic rickets (XLHR) is a rare dominant hereditary bone disorder characterized by excessive renal phosphate excretion and impaired bone mineralization [1]. Patients with XLHR present with hypophosphatemia, short stature, and osteomalacia, with a characteristic “bowing” of the femur [1]. They may also present with enthesopathy and excessive tooth decay. Plasma calcitriol levels are often paradoxically decreased, with preserved calcidiol levels, suggesting impaired vitamin D synthesis. Medical treatment of XLHR involves high dose oral phosphate. Initially, phosphate repletion leads to hypocalcemia and further calcitriol depletion, causing secondary hyperparathyroidism (HPT) and worsening bone disease. Oral calcitriol is given to counteract secondary HPT. However, excessive doses of calcitriol may cause hypercalciuria and nephrocalcinosis. Persistent secondary HPT may also lead to tertiary (autonomous) HPT, with associated symptomatic hypercalcemia [2,3]. The preoperative assessment and surgical management of tertiary HPT associated with XLHR has not been well characterized. We describe the preoperative localization and use of intraoperative quick PTH (IOPTH) to guide successful subtotal parathyroidectomy in a case of XLHR-associated tertiary HPT.

## Case presentation

A 23 year old Caucasian American female with a known diagnosis of XLHR presented with symptomatic hypercalcemia and tertiary HPT. As a child, she had been treated with high dose phosphate and calcitriol supplementation which resulted in renal insufficiency and subsequent hypertension. Recently, she developed bone pain in her back and lower extremities, with increasing fatigue. She was found to have a calcium level of 10.7 mg/dl (normal range 8.5-10.5) with a PTH of 997 pg/ml (normal range 12-65) and was diagnosed with tertiary HPT.

Additional past medical history was significant for a left femoral osteotomy performed for bowing deformity at the age of eight. She underwent a Cesarean section when she was 20, and her pregnancy was complicated by pre-eclampsia. She had no prior fractures or kidney stones.

**Figure 1.**
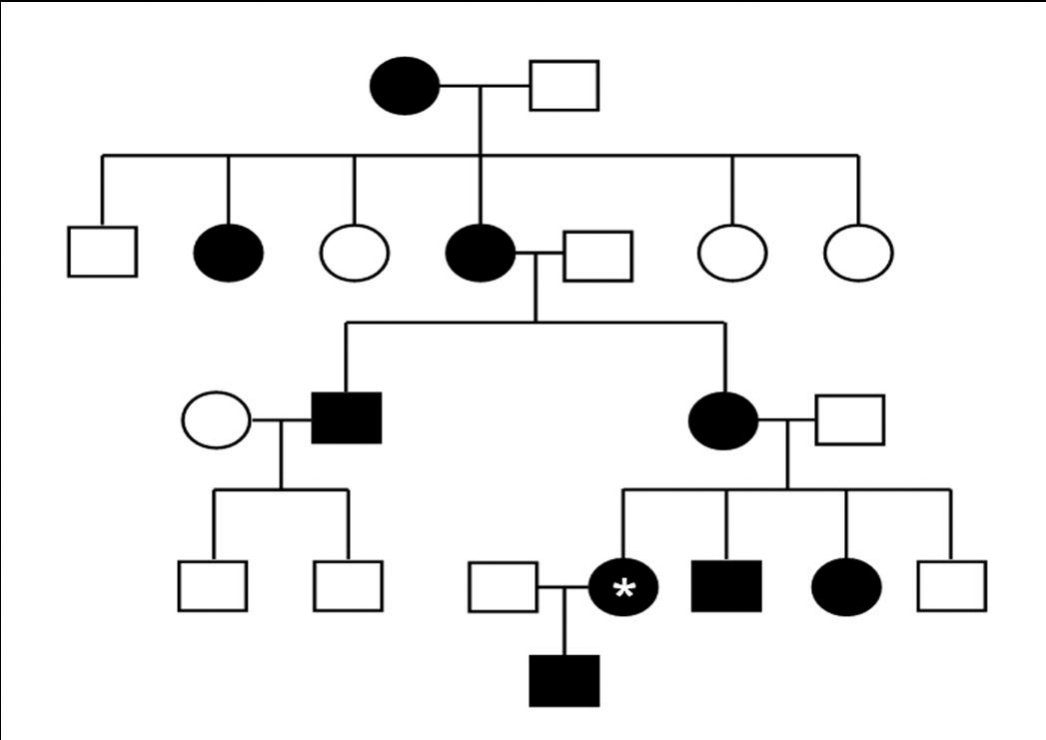
Pedigree representing the dominant X-linked inheritance pattern of XLHR. Our patient is denoted by the asterix (*). Note that neither offspring of the patient’s paternal uncle who carries the trait are affected.

Her medications included labetalol, losartan and 500 mg oral phosphorus. She had been taking 0.25 mcg calcitriol daily, which was recently discontinued. Her family history was significant for multiple family members with XLHR (Figure 1), including her 3 year old son.

On physical examination, she was 162.5 cm in height, 85.3 kg (BMI 31.5). Heart rate was 72 beats per minutes and blood pressure was 150/110 mmHg. She had no band keratopathy. Her thyroid was normal in size with no dominant nodules and she had no cervical lymphadenopathy. She had bilateral bowing of the lower extremities. Serum calcium and PTH levels were 10.7 mg/dl and 1126 pg/ml, respectively. The remainder of her laboratory values were within normal limits including phosphate 2.7 mg/dl, 25-hydroxyvitamin D 25 pg/ml, 1, 25-dihydroxyvitamin D 22 pg/ml, TSH 3.161 µIU/ml, and T4 7.8 µg/dl. A recent bone density study demonstrated low normal values of bone density for her age.

**Figure 2.**
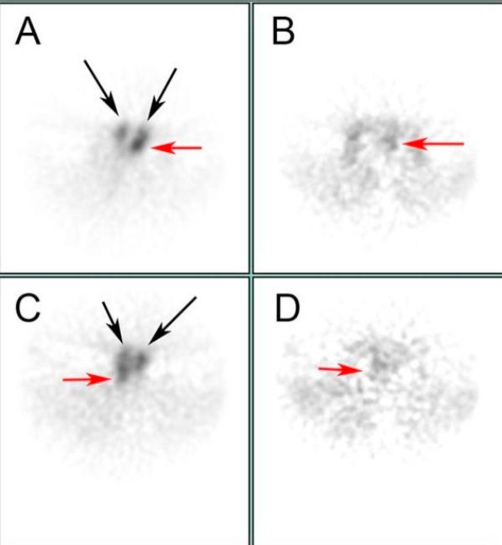
Technetium-99 m Sestamibi SPECT imaging demonstrates focal uptake of tracer in bilateral tracheoesophageal grooves, consistent with bilateral superior parathyroid gland enlargement. **(A)** and **(C)** are represent slices through the left TE groove and more caudal right TE groove parathyroids, respectively. **(B)** and **(D)** are matching late images.

**Figure 3.**
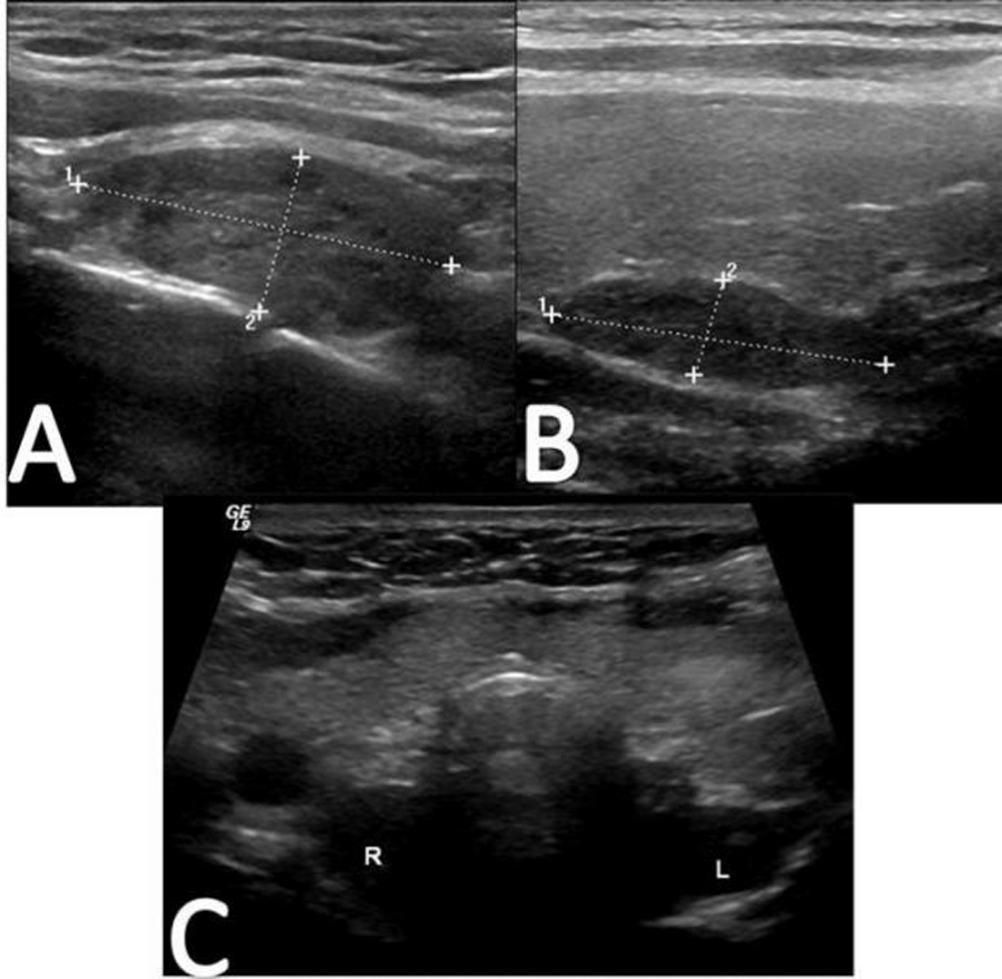
Representative images of the ultrasound of the thyroid and parathyroid glands are shown. **(A)** demonstrates a sagittal view of the right lobe of the thyroid, and a mass measuring 2.93 × 1.23 cm is demarcated by lines 1 and 2 is noted posteriorly in the tracheoesophageal groove. **(B)** demonstrates a 2.63 × 0.69 cm mass in tracheoesophageal groove posterior to the left lobe of the thyroid. **(C)** shows a transverse view of the entire thyroid gland, with the hypoechoic masses noted posteriorly (R,L).

Preoperative evaluation included Technetium-99 m Sestamibi SPECT imaging as well as a complete thyroid and parathyroid ultrasound. The Sestamibi scan showed focal intense tracer uptake on delayed images in the right tracheoesophageal groove, as well as increased tracer uptake suggestive of an enlarged left sided gland (Figure 2). Ultrasound revealed a normal appearing thyroid gland, with bilateral enlarged superior parathyroid glands in the tracheoesophageal groove. Neither inferior parathyroid gland was visualized (Figure 3).

**Figure 4.**
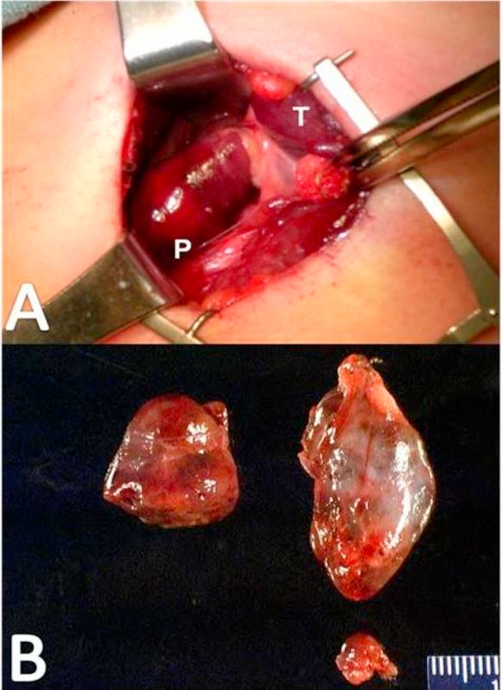
**(A)** Intraoperative photograph demonstrating the enlarged right side superior parathyroid gland (P). Normal appearing thyroid tissue (T) is retracted medially for exposure. **(B)** Appearance of the specimens sent for pathological examination. Note the enlarged bilateral superior glands and the smaller inferior gland. Only one inferior gland was sent for permanent section; the remaining gland was divided in half, with one half left in situ and the other sent for cryopreservation.

A bilateral parathyroid exploration with subtotal parathyroidectomy was planned. Baseline IOPTH was 1298 pg/ml (normal <65 pg/ml). Intraoperative findings correlated with preoperative imaging, with two large superior parathyroid glands and moderately enlarged inferior parathyroid glands (Figure 4). Approximately 100 mg parathyroid tissue was sent for cryopreservation, and approximately 40 mg of the most normal-appearing lower parathyroid was left as an intact remnant. Ten minutes after removal of the last specimen, the PTH level was 114 pg/ml. Of note, the surgical pathology report (supplemental figure) describes all four parathyroid glands as “enlarged and hypercellular”.

As anticipated, she developed symptomatic postoperative hypocalcemia and was initially maintained on an intravenous calcium drip. Her calcium level nadir was 5.7 mg/dl, but slowly corrected over 72 hours to 8.7 mg/dl. She was also treated with oral calcium (2 g calcium carbonate four times daily for 72 hours, then three times daily) and vitamin D supplementation (calcitriol 0.75 mcg daily). She was discharged on postoperative day six with no symptomatic hypocalcemia. Two weeks post-operatively, her calcium level was 9.3 mg/dl, and she had complete resolution of all preoperative symptoms. At follow-up both six and ten months later she remained asymptomatic and her calcium and phosphate levels were normal (9.0 and 2.8 mg/dL, respectively), suggesting no recurrence of tertiary hyperparathyroidism. She had a mild elevation of her PTH (136 mg/dL) in the setting of chronic renal insufficiency (creatinine 1.9).

## Discussion

XLHR is a rare dominant hereditary disorder which occurs in approximately 1 per 20,000 individuals. The pathogenesis of this disease is not fully understood. Cross-transplantation studies using a mouse model (Hyp mouse) demonstrate that the primary phosphate transport defect results from a circulating factor that is extrinsic to the kidney [4]. Thought to be a phosphatonin, this factor promotes phosphate excretion by causing underexpression of the renal sodium phosphate cotransporter [5]. In several forms of hypophosphatemic rickets, similar phosphaturic factors such as fibroblast growth factor 23 (FGF23), frizzled-related protein 4 (FRP 4), matrix extracellular phosphoglycoprotein (MEPE), FGF-7, and stanniocalcin 1, 2 (STC1, 2) have been identified [6,7]. In XLHR, mutations in the osteoblast phosphate regulating endopeptidase gene on the X chromosome (PHEX) lead to failure to inactivate a phosphaturic factor resulting in the clinical sequelae of hypophosphatemia and hyperphosphaturia, slow growth, bone deformities, muscle weakness, and paresthesias [8,9]. Unfortunately, chronic supplementation with phosphorus and vitamin D often results in hypocalcemia, leading to secondary HPT. In the presence of renal impairment, the parathyroids can become autonomously functional, leading to tertiary HPT and hypercalcemia. In these cases, surgical exploration is the only alternative for control of HPT and subsequent hypercalcemic symptoms [10].

The literature regarding the optimal surgical treatment of tertiary HPT is controversial. Although resection of single or double adenoma has been found to be effective in some cases [11], many authors suggest that subtotal parathyroidectomy is required for operative cure [12,13]. Additionally, the rate of recurrence appears to be lower with subtotal parathyroidectomy [14]. Importantly, a thorough review of the literature reveals a paucity of data regarding surgical management of tertiary HPT associated with XLHR. The largest series was a retrospective study containing six patients with different surgical approaches (2-, and 3-gland, total parathyroidectomy), and neither the preoperative evaluation nor the use of IOPTH was addressed. In addition, less than subtotal parathyroidectomy led to recurrence of HPT in one patient [10].

The role of preoperative parathyroid localization including Technetium-99 m Sestamibi SPECT imaging and parathyroid ultrasound in tertiary HPT is unclear, and its efficacy has been questioned in recent studies [15,16]. Sestamibi scans are twice as likely to identify single or double parathyroid adenomas as multiple gland hyperplasia, which makes high-resolution ultrasonography an excellent complementary study [17,18]. In our case, bilateral dominant superior enlarged glands were correctly identified using both Sestamibi and ultrasound. Imaging was also able to exclude supernumerary ectopic glands. The smaller hyperplastic inferior parathyroid glands were not identified by preoperative imaging, confirming the necessity of bilateral, four-gland exploration and subtotal parathyroidectomy.

Although the use of intraoperative PTH is widely established in primary HPT, guidelines for its use in secondary and tertiary HPT remain a source of debate. In primary HPT because of the short half-life of PTH (about four minutes), it has been established that an IOPTH decrease of >50% and into the normal range post-resection is predictive of operative cure [11]. Further, recent studies indicate IOPTH is highly predictive of successful surgical correction of secondary and tertiary HPT, particularly in the setting of renal impairment [19,20]. However, there is a lack of evidence for its applicability in subtotal parathyroidectomy in patients with tertiary HPT associated with XHLR. In our case, we documented a 90% drop in PTH after subtotal resection with normal calcium levels ten months post-operatively, indicating that she was surgically cured of her tertiary hyperparathyroidism, The mild elevation of her PTH was likely reflective of secondary hyperparathyroidism due to chronic renal insufficiency or vitamin D deficiency.

## Conclusion

XLHR is a rare hereditary disease characterized by phosphate wasting, impaired bone mineralization and secondary HPT that may progress to tertiary HPT with symptomatic hypercalcemia. We present the first case of XLHR-associated tertiary HPT using preoperative parathyroid localization and IOPTH monitoring to guide successful subtotal parathyroidectomy. Although this is a rare condition, appropriately guided surgical intervention provides durable cure of HPT and significant symptom relief.

## Abbreviations

HPT: hyperparathyroidism
IOPTH: intraoperative quick PTH
PTH: parathyroid hormone
XLHR: X-linked hypophosphatemic rickets
